# Water shortage: Assessment and analysis on a regional scale

**DOI:** 10.4102/jamba.v16i1.1649

**Published:** 2024-10-11

**Authors:** Yuri M. Macedo, Jhonathan L. de Souza, Adriano L. Troleis

**Affiliations:** 1 Department of Geography, Instituto Federal do Rio Grande do Norte, Macau, Brazil; 2 Institute of Geosciences, State University of Campinas, Campinas, Brazil; 3 Department of Geography, Federal University of Rio Grande do Norte, Natal, Brazil

**Keywords:** risk of water shortage, disasters, risk assessment, Rio Grande do Norte, Brazilian semi-arid

## Abstract

This paper, adopting theoretical-methodological assumptions, aims to analyse the risk of municipal urban water shortage in the state of Rio Grande do Norte (RN), Brazil, through the results of the Water Shortage Risk Index (WSRI). The WSRI is the product of the analysis of 19 variables, in a systemic perspective that involves environmental, infrastructural, socioeconomic and state planning indicators. The survey was carried out in the 153 municipalities that make up the water supply system managed by the state concessionaire (representing 92% of the 167 municipalities in the State), in its seven water supply regions. The WSRI result identified 49.0% of the municipalities analysed in the ‘high’ and ‘very high’ risk classes; 40.5% as ‘medium’ risk and 10.0% as ‘low’ risk, with no occurrences of ‘very low’ risk. In absolute values, 1 municipality was classified as ‘very high’; 74 were classified as ‘high’; 62 as ‘average’; and 16 were considered to be at ‘low’ risk of water shortages.

**Contribution:**

To decrease and/or mitigate the results of the WSRI in the State, the transposition of basins, integration of supply systems, hydrogeological research, among others, were proposed.

## Introduction

Water scarcity is a problem in several regions of the world, affecting millions of people on all continents, as shown by the research conducted by Tzanakakis, Paranychianakis and Angelakis (2020): (1) More than 2 billion people live in regions with high water stress and this number is expected to increase; (2) more than 1bn people do not have access to clean and safe drinking water; (3) about 3.4 million people die each year from using contaminated water; (4) millions of women and children spend several hours a day collecting water over an average distance of 6 km; (5) at any given time, half of the world’s hospital beds are occupied by patients suffering from diseases associated with the lack of access or poor quality of drinking water. Specifically, water shortage is characterised as a disaster for promoting:

[*A*] grave interruption on the functioning of a society, causing general human, material, economic or environmental losses that exceed the capacity of the affected society of dealing with its own resources. (UNESCO 2010)

Water shortages in the Brazilian semi-arid region are a recurring problem with great socioeconomic impact, since it is the most populous semi-arid region in the world (Ab’saber 1999). The state of Rio Grande do Norte (RN) has most of its territory located in the semi-arid region where, because of the last hydrological drought, between 2012 and the first quarter of 2019, in this context, the Government renewed the water emergency decree for another 12 months of the year, that is, legal regulation covers around 152 of its 167 municipalities, corresponding to 91% of the entire state territory. Related to this, in March 2017, 23 municipalities in the state were identified in a situation of water collapse by the state water supply concessionaire (CAERN).

In this context, this research proposes a methodology for the assessment and analysis of the risk of water shortages applied in the state of RN, Brazil. The research was conducted from the perspective of disaster risk reduction (DRR), in line with the goals of the Sendai Framework for DRR 2015–2030 built by the United Nations International Strategy for Disaster Reduction (UNISDR 2015). Therefore, it brings the understanding and evaluation of this type of risk together with proposals for its reduction, based on the actions recommended in the Sendai Framework.

The use of a system of indicators, with its categorised and weighted variables, resulting in a synthesis index, was based on studies done by Medeiros (2014, 2018); Macedo (2015); Welle and Birkmann (2015); Almeida, Welle and Birkmann (2016); Oliveira (2018); and Macedo, Troleis and França (2020), Macedo, Troleis and Almeida (2021). Therefore, the production of indices has become a fundamental methodology in disaster risk assessment studies. Some indices on the subject of water resources stand out, such as the Water Stress Index (WSI) (Falkenmark & Lindh 1974) as ‘one of the first integrated assessments between water resources and population at the Third World Population Conference in Bucharest in 1974’ (Damkjaer & Taylor 2017). Subsequently, the work of organisations such as the Intergovernmental Panel on Climate Change (IPCC 2007, 2012) and the Aqueduct 3.0 platform of the World Resources Institute (WRI) were produced:

[*W*]hich analyzed several hydrological models and calculated the amount of water withdrawn from surface supplies and groundwater available in each region of the world compared to the total available water. (Cajazeiras 2020)

In Brazil, the works on the subject of water resources follow the perspective of climate vulnerability to the phenomenon of drought, produced by the National Institute for Space Research (INPE 2007, 2015), and the indicators of vulnerability to drought (Diniz 2019; Rosendo 2014; Rosendo et al. 2017). Furthermore, Castro’s (2010) thesis is inserted directly into the theme of water supply, which brings a functional analysis of the infrastructure and quality of metropolitan water supply in Rio de Janeiro. Specifically in the study area of this article, RN, there is the work of Troleis and Silva (2018b, 2019) in which they proposed a methodology of territorial vulnerability to the collapse of water supply.

In comparison to the works cited, this article is distinctive by the scale of analysis, methodological proposal and focus of analysis. In this research, the water availability of the underground spring, its vulnerability to contamination and the capacity of municipal urban water storage are some variables among others that make up the IRDH. In this context, the proposed risk index presents variables in an integrated manner, related to the natural environment, technical infrastructure objects, socioeconomic characteristics and institutional planning.

Therefore, the main objective of this work is to assess the risk of water scarcity on a regional scale based on the Water Scarcity Risk Index (WSRI). To this end, its specific objectives were: (1) to present the WSRI as a system of socio-environmental indicators of water scarcity; (2) analyse regional patterns of WSRI spatialisation.

## Research methods and design

### Field of study

This research study comprises of 153 cities of RN supplied by the concessionaire of water supply of Rio Grande do Norte (CAERN). According to the last census of the Brazilian Institute of Geography and Statistics (IBGE 2010b), the total urban population of these cities is 2 320 149 inhabitants. The remaining 14 cities of RN, which are supplied by autonomous systems ministered by each city hall, totalling 167 cities in the State, were not included in this analysis because of the absence or precariousness of availability of data.

**FIGURE 1:**
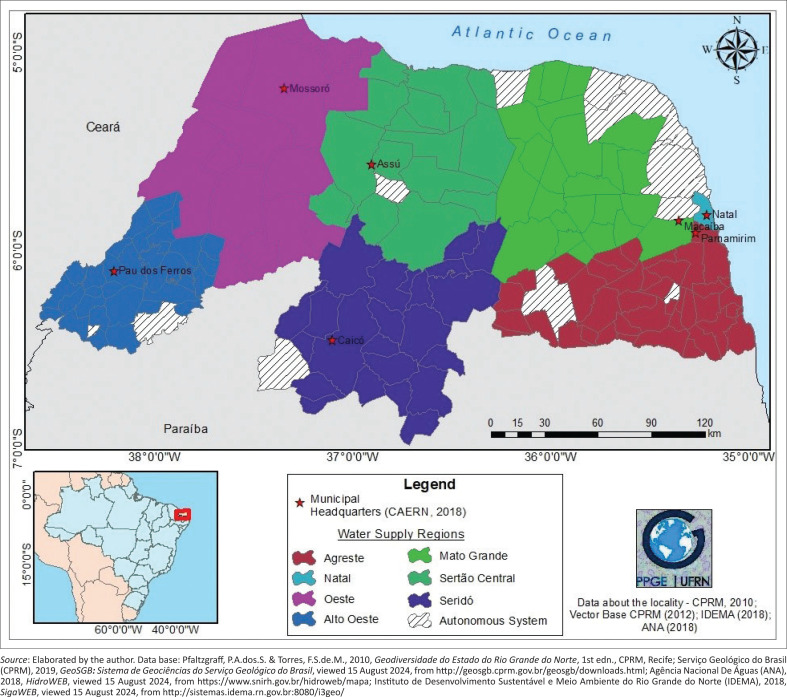
Location of Rio Grande do Norte and regionalisation of the water supply system.

Regarding the natural characteristics of the State, RN presents a semi-arid climate in the largest part of the territory (Diniz & Pereira 2015), which brings 7 to 8 months per year of dry weather, with a rainfall average of 600 mm/year (Figure 1). The predominant semi-arid climate in the state contributed to, between 2012 and 2017, the occurrence of the longest period of dry weather registered in the last 100 years.

Besides the climate condition, the State’s countryside is located, in its largest part, above the foundation of igneous and metamorphic crystalline rocks. This limits the access to the underground water by the population because of the characteristics of low availability and quality of the fissure aquifer, classified as ‘very low’ in regard to the hydro and geologic potentiality (Medeiros, Nascimento & Sousa 2010), affecting directly the municipal urban water supply.

Relating the climate with the geology of RN, the most important elements of the natural dynamic for the WSRI, can be classified as the most vulnerable units to this disaster. For example, the Potiguar crystalline aquifer associated to the semi-arid climate increases the natural vulnerability to water shortage, which hinders the underground (in quantity and quality) and superficial water usage because of the low volume of rain in 10 months of drought, an inherent behaviour of this type of climate. In turn, the semi-arid climate in the East coast of the State, associated with the Barreiras aquifers (sedimentary basin), increases the possibilities of water capture, both in the underground and superficial fountainhead, because of the high level of rainfall (more than 1000 mm of annual average); the permeability and capacity of storage of the Barreiras aquifer also reduce the vulnerability to water shortage in this region of the State.

### Methodological procedures

Conceptually, this article discusses risks of disasters (risk of water shortage), understood as a function between danger and vulnerability. Hence, the concepts of risk, danger and vulnerability may be defined as starting point for the theoretical and methodological conception of this work.

The concept of risk used in this article is defined through a function between social vulnerability and the natural dangers of a local: *R* = f (P, V) – *R* = Risk; *P* = Danger; *V* = Vulnerability. According to Veyret (2007), risk is a social construction and it is directly connected to the conception of the population with regard to some potential danger of physical damages and/or material loss of great amount. Regarding vulnerability, it results from the capacity of each individual when it comes to ‘the resistance or capacity of dealing and recovering oneself from the impact of a natural danger’ (Blaikie et al. 1994:9). In this perspective, the vulnerability is understood as a condition of susceptibility to some event that potentially causes physical and material damages to the resident population.

As such, risk can be understood from a socio-environmental perspective, as a socio-environmental vulnerability (Alves 2006; Esteves 2011; Freitas et al. 2012; Girão, Rabelo & Zanella 2018; Macedo 2015 2018; Mendonça & Leitão 2008; Zanella et al. 2013).

Risk is a social construction, and is directly linked to the population’s conception of any potential danger of causing physical damage and major material losses. A population may not even realise that they are at risk. In this research, among the types of environmental risk, ‘natural risks aggravated by man’ is the one that best fits, because, as classified by Veyret (2007:24), it is the ‘result of a natural hazard whose impact is expanded by human activities and the occupation of the territory: erosion, desertification, fires, pollution, floods, etc.’

In turn, danger would be the agent causing material and/or immaterial damage or losses. This article adopts the concept of danger as used by Smith (2001), being ‘an ineluctable part of life and a potential threat to people and their property, while risk is the probability of a danger occurring and generating losses’ (Smith 2001:392).

Vulnerability would be the measurement of each individual’s ability to prepare, cope, resist and have resilience when exposed to a danger. From this perspective, vulnerability is understood as a condition of susceptibility to some event potentially causing material and physical damage to the resident population. According to Blaikie et al. (1994):

‘By vulnerability we mean the characteristics of a person or group in terms of their ability to predict, deal with, resist and recover from the impact of a natural hazard. It is a combination of factors that determine the degree to which someone’s life and livelihood are put at risk by a discrete and identifiable event in nature or society’. (Blaikie et al. 1994:9)

Detailing the relationship between risk and vulnerability, this research follows the conception of Almeida et al. (2016), in which risk is composed of two dimensions, one natural and the other social. The first comprises exposure to natural hazards that trigger a disaster, while the second is related to vulnerability and has three central components: susceptibility, ability to cope, and adaptability.

In this regard, the environmental, state planning, and social and economic indicators that compose the WSRI were defined through the studies of risk of disaster such as the IPCC (2012), UNISDR (2009); Macedo (2015); Medeiros (2018); Oliveira (2018), as well as through the propositions of managers and technicians connected to the water supply of the State. They allowed for the production of a systematic index that comprised 19 variables inherent to the exposition, capacity of coping and adaptation of the population of the studied region in what concerns the municipal urban water shortage in RN.

In this context, the reference disaster risk analysis and assessment methodologies for this research have in common the production and analysis of a system of indicators, with its variables categorised and weighted, resulting in a synthetic index. On a global scale, there is the Welle and Birkmann’s (2015) World Risk Index. At the national level, Almeida et al.’s (2016) DRIB (Disaster Risk Indicators In Brazil). Analysing locally, at the state and/or municipal level, the works of Medeiros (2014, 2018); Macedo (2015); Oliveira (2018); and Macedo et al. (2020, 2021) stand out.

The definition of weights for each variable and indicator was defined by the researcher and advisor based on several simulations that were adjusted from a questionnaire applied to engineers and technicians of the CAERN, that is responsible for the regional water supply system of the state. Each variable was categorised into 5 risk levels, where 1 represents the lowest risk of the category within the variable and 5 the highest. Each variable was ranked within the indicator, which, in turn, was weighted within the index, so that the sum of the weights of each variable and/or indicator is equal to the number of variables and/or indicators.

After establishing the weight of each variable and indicator in the sample, the weighted arithmetic average was applied, which allowed for two indexes, one that specifically represents the Average per Indicator (MI), and another one that represents the general result of the WSRI. The acquisition of two indexes can be identified in the Equations 1 and 2. In Equation 1, we have the MI:


$$MI=\frac{\sum_{i}^{n}\left(V_{i}\cdot PV_{i}\right)}{\sum_{i}^{n}PV_{i}}$$ [Eqn 1]

Where:

*i* = First variable

*n* = Last variable

*V*_*i*_ = Result obtained for the ith variable of a specific indicator

*PV*_*i*_ = Weight that refers to the respective variable obtained in the data collection of the research.

The MI has the goal of establishing an index, giving a diagnostic of the situation of the referring city to each indicator. Furthermore, this value will be used to determine the general index of the WSRI, using Equation 2:


$$IRDH=\frac{\sum_{j}^{n}\left(MI_{j}\cdot PI_{j}\right)}{4}$$ [Eqn 2]

Where:

*j* = First indicator

*n* = Last indicator

*MI*_*j*_ = Result of the Equation 1 for the jth indicator

*PI*_*j*_ = Specific weight of the respective indicators.

The result of the WSRI consolidates in a single parameter the risk of water shortage in each city, since it considers all of the variables and indicators collected in the study. Another important aspect in the composition of the index was the categorisation of data of each variable in five categories: level 1 – very low risk; level 2 – low risk; level 3 – medium risk; level 4 – high risk; and level 5 – very high risk.

The process of spatialisation of the WSRI was possible through the urban city data collection referring to the indicators that compose the index (Environmental; Infrastructural; State Planning; and Social and Economic) attached to the city polygonal georeferenced in a System of Geographical Information (S.I.G.) environment, which allowed for the production and analysis of the WSRI results. In this context, it is necessary to detail the indicators and its variables, its weighting and basis. The variables and weighting correspond to indicators: Environmental, Infrastructural, State Planning, and Social and Economical – where, PV is weight of the variable and PI is weight of the indicator. All variables were categorised into levels from 1 to 5 (N1, N2…) and tabulated in the calculation of the index.

The environmental indicator, with the second highest weight inside the WSRI, had a value of 1.0. Its importance resides, especially, in the exposition and susceptibility of cities to water shortage, and, therefore, affects directly the increase or reduction of the municipal risk for this disaster. The variables that compose this indicator are, in order of importance in the weighting:

- V1 – Average outflow of the wells – (Weight 1.25) and the categorisation: superior to 50.1 (N1); 25.1 to 50.0 m^3^/H (N2); 10.1 to 25.0 m^3^/H (N3); 5.1 to 10.0 m^3^/H (N4); 0.1 to 5.0 m^3^/H (N5). The underground spring is a fundamental alternative to water supply; it is present in state sanitation plans, such as those in RN (SERHID 1998) and Ceará (SRHCE 2018), with data on wells and flow (inventory), as well as the survey by the Geological Survey of Brazil (Beltrão et al. 2005). The data were obtained from the database of the State Secretariat for the Environment and Water Resources (SEMARH 2019).
- V2 – Natural superficial water bodies in the city (Weight 1.25) with the categorisation: perennial river (N1); perennial stream (N2); intermittent river (N3); intermittent stream (N4); absence of water bodies in the city (N5). They are also an alternative for supply and are present in state and river basin sanitation plans. The data were obtained from the SEMARH database (2019), the State Institute for the Environment (IDEMA 2018) and the National Water Agency (ANA 2018), which were researched in a Geographic Information System (GIS) environment.
- V3 – Predominant climate type in the city (Weight 0.75) and the categorisation: humid tropical (N1); semi-humid tropical (N2); mild semi-arid tropical (N3); medium semi-arid tropical (N4); strong semi-arid tropical (N5). Climate is fundamental to understanding territorial dynamics in terms of water availability. The data are available in the state and river basin sanitation plans; surveys by CAERN, SEMARH and the Rio Grande do Norte Water Management Institute (IGARN), among others, which use climate data in their monitoring for water resources management. The data were obtained from the classification and map generated by Diniz and Pereira (2015).
- V4 – General vulnerability of the underground fountainhead of municipal water supply (Weight 0.75) and the categorisation: imperceptible (N1); low (N2); medium (N3); high (N4); extreme (N5). It has indirect importance in the environmental indicator, influencing the contamination of underground water sources. Data of this nature are found in materials specialised in hydrogeology, such as the hydrogeology manual of the Geological Survey of Brazil (Feitosa et al. 2008). To obtain this data, an analysis was carried out using the Groundwater Hydraulic confinement - Overlaying strata - Depth to groundwater table (GOD) (Foster & Hirata 1988). They are also an alternative for supply and are present in state and river basin sanitation plans. The data were obtained from the SEMARH database (2019), the State Institute for the Environment (IDEMA 2018) and the National Water Agency (ANA 2018), which were researched in a GIS environment.

In turn, there is the infrastructural indicator, with the highest weight of the WSRI (1.75). The variables that compose this indicator are, in order of importance in the weighting:

- V5 – Capture type (Weight 1.5) and the categorisation: supplied by wells of the Potiguar sedimentary basin (granular) (N1); mixed supply – wells and water supply networks (N1); supplied by water supply networks in the Potiguar sedimentary basin (N2); supplied by networks with capture in dams (or other fountainheads) in the Potiguar crystalline (N3); supplied by wells in the Jandaíra aquifer (N3); supplied by wells of the crystalline (N4); supplied by large municipal dams (local) in the Potiguar crystalline (N4); supplied by small dams, with capacity of up to municipal (local) 25 million of m^3^ (or other captures) in the Potiguar crystalline (N5). This variable is fundamental within the IRDH indicator system. Capture possibilities are present in state and municipal sanitation plans, in addition to diagnoses of various water resources such as that of the National Water Agency (ANA 2014) and the state agency (SEMARH 2014). The data were obtained by crossing municipal water quality reports that contain the type of supply (CAERN 2018, 2019), associated with the questionnaire applied to the Seridó region of CAERN.
- V6 – Capacity of the cities’ reservoirs (Weight 1.0) and the categorisation: reservoir(s) with more than 600 m^3^ of capacity (N1); reservoir(s) between 451 m^3^ and 600 m^3^ of capacity (N2); reservoir(s) between 251 m^3^ and 450 m^3^ of capacity (N3); reservoir(s) with capacity between 50 m^3^ and 250 m^3^ (N4); reservoir smaller than 50m^3^ of capacity or absence of reservoirs and/or direct distribution in the network (N5). Water storage equipment is essential for dealing with and resisting water collapse or intermittency events (Del Grande 2016). This information is found in municipal sanitation plans, in the diagnosis phase (PMBJ 2017). The data were obtained through a questionnaire applied to the Seridó region of CAERN.
- V7 – Time of installation of the urban water supply network (Weight 1.0) and the categorisation: network installed in up to 10 years (N1); network installed from 10 to 15 years (N2); network installed from 15 to 20 years (N3); network installed from 20 to 30 years (N4); network installed in more than 30 years (N5). This variable is a parameter as an indicator of the vulnerability of the supply system. Time promotes wear on the structure. This information can also be found in municipal sanitation plans. The data were obtained through a questionnaire applied to the Seridó region of CAERN. Water storage equipment is essential for dealing with and resisting water collapse or intermittency events (Del Grande 2016). This information is found in municipal sanitation plans, in the diagnosis phase (PMBJ 2017). The data were obtained through a questionnaire applied to the Seridó region of CAERN.
- V8 – Constructive material of the network of water supply (Weight 1.0) and the categorisation: PVC tube (N1); PVC tube and molten iron (N2); molten iron (N3); PVC tube or molten iron and asbestos cement (N4); asbestos cement (N5). It is an indicator of the vulnerability of the supply system. This information was not found in any other reference. The data were obtained through a questionnaire applied to the Seridó region of CAERN.
- V9 – Existence of sewage treatment and waste collection in the city (Weight 0.75) and the categorisation: collection of weekly and/or regular urban residues (landfills) and sewage treatment (N1); sewage treatment with absence of collection of urban residues and/or destination to landfills (N3); collection of weekly and/or regular waste and destination to landfills without sewage treatment (N4); absence of sewage treatment and no ideal destination for urban residues (N5). Waste disposal and sewage are agents of water source pollution, which can make water supply unviable. It is present in state sanitation or water resources plans such as the one in RN (SERHID 1998). The data were obtained from a questionnaire applied to the CAERN region and cross-checked with the National Sanitation Information System (Brasil 2019) and the National Basic Sanitation Survey (IBGE 2017b).
- V10 – Percentage of households supplied by piped water in an urban environment (Weight 0.75) and the categorisation: 90% to 100% (N1); 75% to 90% (N2); 50% to 75% (N3); 25% to 50% (N4); up to 25% (N5). It is an indication of the lack of infrastructure in the supply network. This information can also be found in municipal and state sanitation plans. It was obtained from data available in the National Sanitation Information System (Brasil 2019).

The indicator State Planning has intermediate weight in the WSRI, with a value of 0.75. The variables that compose this indicator are:

- V11 – Occurrences registered in the collapse of the city’s urban supply in the last 6 years of drought (Weight 1.5) and the categorisation: no collapse of municipal urban water supply in the period between 2012 and 2017 (N2); partial collapse in the municipal urban water supply or in the system of rotation in the period between 2012 and 2017 (N3); collapse of the municipal urban water supply in the period between 2011 and 2017 (N5). This variable is important as an indicator of the vulnerability and exposure of one municipality in relation to another. This information is not found in common references, being obtained from a questionnaire applied to the CAERN regional office and cross-checked with the RN supply chart (CAERN 2018, 2019).
- V12 – Existence of a municipal plan referring to the water supply (Weight Variable 1.0) and the categorisation: it exists (N2); it is in planning (N3); it does not exist (N4). The municipal sanitation or water resources plan is a fundamental planning instrument to reduce the risk of disasters of this type. The data for this variable were obtained from the section ‘MUNIC – General aspects of basic sanitation policy management’ (IBGE 2017c).
- V13 – Structural measures for the reduction of risk of shortage (Weight 0.75) and the categorisation: existence of structural construction works of reservoirs or distribution networks (N2); existence of a project with allocated resources for structural construction works of the municipal Serviço Autonomo de Águas e Esgoto (SAAE) (N3); existence of another option for water supply in case of collapse (N4); absence of options for supply (N5). Chosen from studies on the risk of disasters such as landslides and floods (Guerra 2009; Macedo 2015; Medeiros 2018), in which ongoing projects or works that improve local infrastructure conditions are analysed with a view to reducing the risk of these disasters; and also in state water resources plans (SERHID 1998; SRHCE 2018). The data were obtained through a questionnaire applied to the Seridó region of CAERN.
- V14 – Risk management of shortage in the city (Weight 0.75) and the categorisation: existence of a secretary of social security, water resources, environment and public security with a structured civil defence (N1); existence of instruments of control and monitoring for DRR of water shortage (N2); existence of civil defence with interinstitutional structure and an articulation (N3); existence of a municipal plan of DRR or contingency plan (N4); existence of civil defence only (or not) without minimal structure (N5). It was also chosen based on disaster risk studies. In the case of water shortages, the municipal water resources, environment and/or sanitation department is also important. This information was obtained from the section ‘MUNIC – Profile of Brazilian Municipalities – Risk Management and Responses to Disasters’ (IBGE 2017a).

The Social and Economic indicator is the one with the lowest weight in the WSRI, with a value of 0.50. The variables that compose this indicator are:

- V15 – Quantity of urban inhabitants of the cities (Weight 1.5) and the categorisation: from 100 to 1000 inhabitants (N1); from 1000 to 2500 (N2); from 2500 to 60 000 inhabitants (N3); from 60 000 to 250 000 inhabitants (N4); more than 250 000 inhabitants (N5). It was chosen from disaster risk studies in Welle and Birkmann (2015) and Almeida et al. (2016). It is a variable that directly affects the population’s exposure to the dangers of disasters; the more inhabitants exposed, the greater the risk. Data for this variable were obtained from the IBGE (2010a).
- V16 – HDIM – Income (income per capita of the population, namely, the average monthly income of the individuals in the city), and V17 – HDIM – Education have weight 1.0 and the categorisation: very high Municipal - Rural Human Development Index (HDIM-R) (N1); high HDIM-R (N2); medium HDIM-R (N3); low HDIM-R (N4); very low HDIM-R (N5). These two variables were chosen from disaster risk studies for the world (Welle and Birkmann 2015) and for Brazil (Almeida et al. 2016). They are an important variable to increase the ability to deal with and adapt to disasters, and data on these variables were obtained from the IBGE (2010a).
- V18 – Level of coverage of income transfer programme (Weight 0.75) and the categorisation: above 75% of the population (N1); 50% to 75% of the population (N2); 25% to 50% of the population (N3); 10% to 25% of the population (N4); up to 10% of the population (N5). It is an important variable for the population to increase its ability to deal with and adapt to disasters. The data for this variable were obtained through the single Bolsa Família register, available for each municipality on its own website, linked to the ministry of citizenship – National Secretariat of Income and Citizenship, with data referring to the year 2019.
- V19 – Gini Index (Weight 0.75) and the categorisation: very low Gini index – 0.0 a 0.19 (N1); low Gini index – 0.20 a 0.39 (N2); medium Gini index – 0.40 a 0.59 (N3); high Gini index – 0.60 a 0.79 (N4); very high Gini index – 0.80 a 1 (N5). It is also an important variable in increasing the ability to deal with and adapt to disasters. The more social equality, the shorter the distance between access to health services, transportation and decent housing, between rich and poor. Data for this variable were obtained from the IBGE (2010a).

In view of the foregoing, it is worth noting that this work did not need to go through ethics committees, given the use of secondary data that does not refer to people or animals.

### Ethical considerations

An application for full ethical approval was submitted to the Federative Republic of Brazil, Ministry of Education, Federal Institute of Education, Science and Technology, Rio Grande do Norte, and an ethics waiver was received on 18 February 2023. The Institutional Review Board issued an ethics waiver for the study as it does not involve any studies with animals or human participants conducted by the authors. Although this study did not involve human participants or animals, all procedures followed were in accordance with institutional and international ethical standards for research integrity and transparency.

## Results

In RN, the regions of water supply, Seridó and Alto Oeste Potiguar, are historically the most problematic when it comes to water safety in the State. In the last period of prolonged dry weather (6 years, between 2012 and 2017), these two regions were the ones that presented the highest quantity of cities in water collapse or supply rotation, according to the information of the situation of supply of the concessionaire (CAERN 2018, 2019).

**FIGURE 2:**
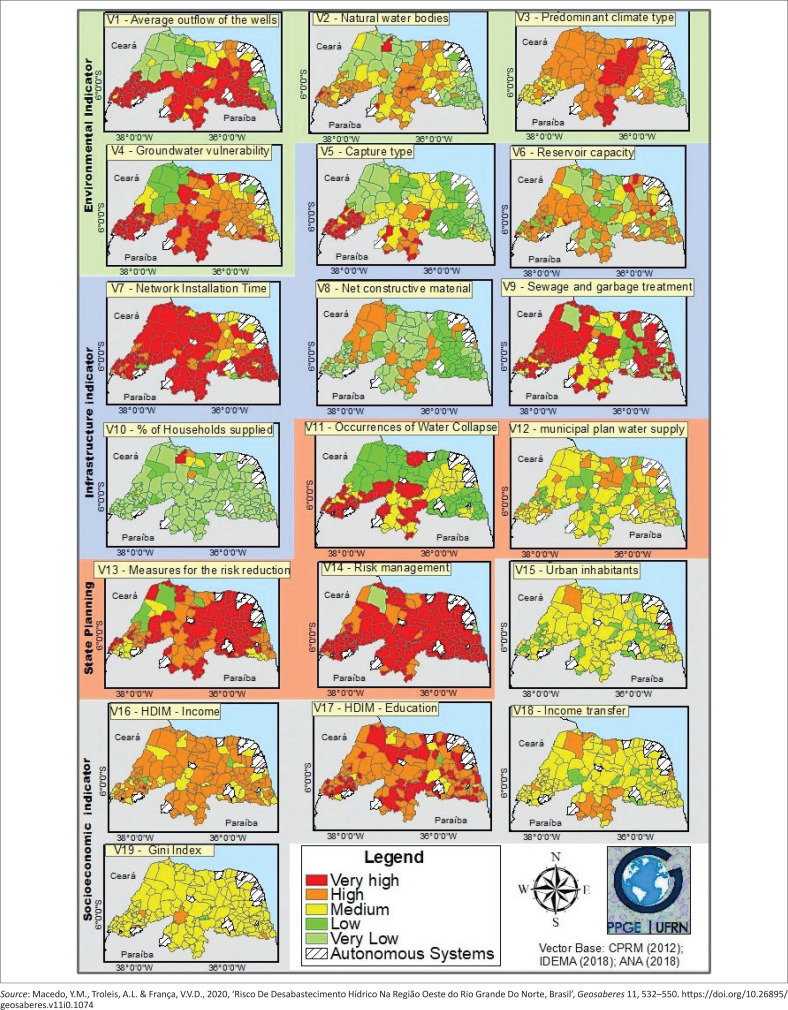
Water Shortage Risk Index – Individualisation of the variables.

Figure 2 shows the result of the 19 variables, organised by indicators. They are, in sequence: variables 1 through 4 – environmental indicator; variables 5 through 10 – infrastructural indicator; variables 11 through 14 – state planning indicator; variables 15 through 19 – social and economic indicator.

**FIGURE 3:**
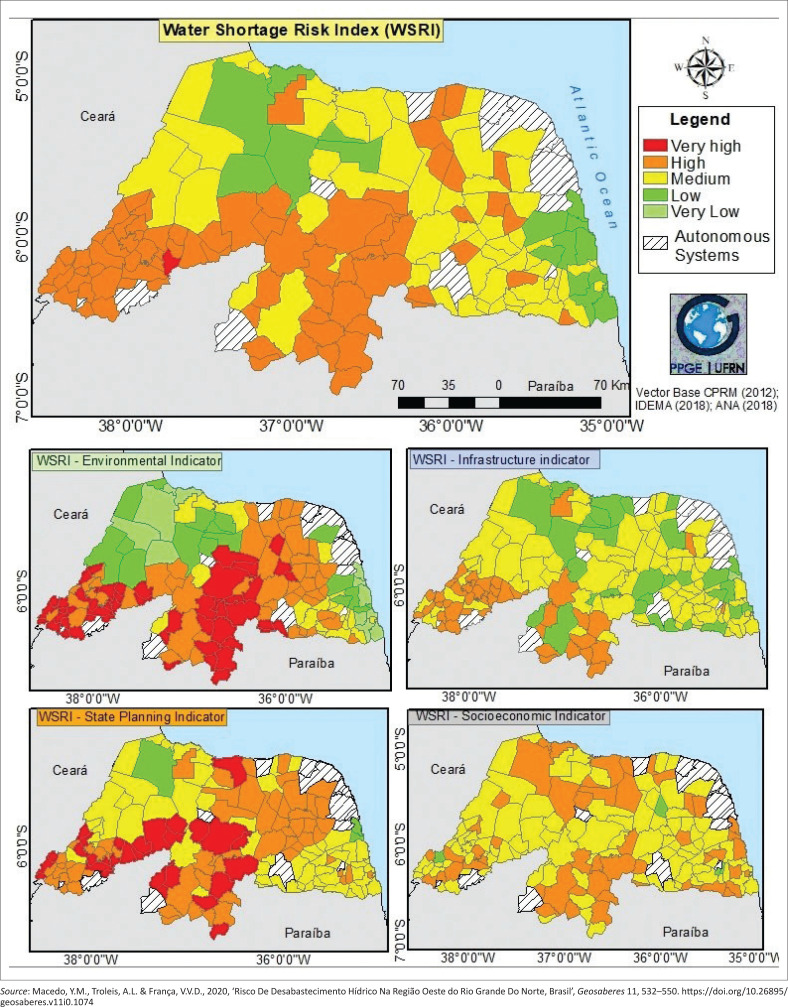
Water Shortage Risk Index and its indicators.

Figure 3 shows the result of the four indicators that make up the index. Regarding the Environmental indicator, specifically, it presented, among the 153 analysed cities, 52 of them at a ‘very high’ risk level (34%) with a total of 307 995 inhabitants (14%); 51 at a ‘high’ risk level (33%), totalling 279 102 inhabitants (12%); 21 at a ‘medium’ risk level (14%) adding up to 136 408 inhabitants (6%); 17 cities at a ‘low’ risk level (11%) totalling 190 088 inhabitants (9%); and, finally, 12 cities at a ‘very low’ risk level (8%) comprising 1 332 633 inhabitants (59%).

In turn, the Infrastructural indicator of the WSRI presented 43 cities with ‘high risk’ (28%), totalling 226 050 inhabitants (10%); 79 cities (51%) with ‘medium’ risk, with a total of 444 661 inhabitants (20%); 30 cities (20%) with ‘low’ risk, with a total of 1 373 059 inhabitants (61%); and only 1 city (1%) with ‘very low’ risk, comprising 202 456 inhabitants (9%). This result can be analysed through the influence of more than a thousand kilometres of water supply networks that permeate RN, bringing water to the cities for up to 315 km of extension.

One example is the case of the Monsenhor Expedito network, which supplies 30 cities and serves approximately 265 040 inhabitants, with main capture in the Bonfim Lake, city of Nisia Floresta. Besides this water supply network, others that stand out are the Alto Oeste system, with its two subsystems (with capture in the Santa Cruz do Apodi and Pau dos Ferros dams), which will supply 27 cities when their total operations initiate. Another important network system is the Sertão Central Cabugi, which serves 8 cities in the central region of the State, capturing water of the Armando Ribeiro Gonçalves dam, and serving approximately 47 527 inhabitants. Generally speaking, 81 cities are supplied by water supply networks, either exclusively or mixed (alongside wells), therefore 53% of the analysed study area and 70% of the population.

The result of State Planning indicator was that the majority of the cities presented ‘medium’ risk in the largest part of the state territory, followed by ‘high’ and ‘very high’ risks, with none being classified as ‘very low’ risk. Figure 3 shows that the State Planning indicator presented ‘very high’ risk in 29 cities (19%), totalling 164 223 inhabitants (7%); 61 cities (40%) were classified as ‘high’ risk, totalling 223 879 inhabitants (10%); 60 cities (39%) with ‘medium’ risk, with a total of 614 688 inhabitants (28%); 3 cities (2%) with ‘low’ risk, totalling 1 243 436 inhabitants (55%); and no city with ‘very low’ risk. It is worth noting, however, that the classification of ‘low’ risk has 3 cities only, albeit it also has the largest number of inhabitants. That is because the most populated cities of the State are in this category, namely, Natal, Mossoró, and Parnamirim.

The Social and Economic indicator result shows that 52 cities (34%) have ‘high’ risk level, totalling 1 624 894 inhabitants (72%), 97 have ‘medium’ risk level (63%) with 613 938 inhabitants (27%), and 4 cities (3%) have ‘low’ risk level, with 7394 inhabitants (1%), approximately. No city was classified with ‘very high’ or ‘very low’ risk. With this, the higher quantity of inhabitants is in the ‘high’ risk class, showing the influence of the variable V15 – quantity of urban inhabitants in which the most populated cities in the State (Natal, Mossoró and Parnamirim) are located. Table 1 briefly shows the results for each index indicator, with the number of people and the total number of municipalities.

**TABLE 1:** Quantitative municipal urban population of the Water Shortage Risk Index by risk level.

Risk level	Indicator	Number of municipalities	Total urban population (in hab.)
Very high	Environmental	52	307 995
	Infrastructure	-	-
	State Planning	29	164 223
	Socioeconomic	-	-
	WSRI-RN	1	3479
High	Environmental	51	279 102
	Infrastructure	43	226 050
	State Planning	61	223 879
	Socioeconomic	52	1 624 894
	WSRI-RN	74	373 702
Medium	Environmental	21	136 408
	Infrastructure	79	444 661
	State Planning	60	614 688
	Socioeconomic	97	613 938
	WSRI-RN	62	426 401
Low	Environmental	17	190 088
	Infrastructure	30	1 373 059
	State Planning	3	1 243 436
	Socioeconomic	4	7394
	WSRI-RN	16	1 442 644
Very low	Environmental	12	1 332 633
	Infrastructure	1	202 456
	State Planning	-	-
	Socioeconomic	-	-
	WSRI-RN	-	-

*Source:* Instituto Brasileiro De Geografia e Estatística (IBGE), 2010a, *Censo demográfico 2010: número de habitantes*, Rio de Janeiro, viewed 15 August 2024, from https://cidades.ibge.gov.br/.

In hab, inhabitants; WSRI-RN - Water Shortage Risk Index of Rio Grande do Norte.

## Discussion

### Water Shortage Risk Index of Rio Grande do Norte

The WSRI-RN presents a relevant result in the research, since, besides drawing attention to the risk of water collapse in the regions of the State, it also proposes a discussion regarding the measures that must be taken when it comes to the integration of systems, research for underground fountainheads, and investments in water infrastructure, such as construction of larger reservoirs, and renovation of the distribution network and municipal basic sanitation.

When compared to other states, the RN has a good water infrastructure, with more than 1000 km of water supply networks distributing treated water mainly to the cities of the semi-arid Potiguar. Even so, a lot of the cities of the State registered a collapse of water supply in the last period of drought, revealing the largest vulnerability of the Environmental indicator, through the predominant climate and geology of the countryside cities, if compared to the coast.

The result of the WSRI-RN did not present a city with a ‘very low’ risk level of shortage. However, 1 city (1% of the cases) was classified as ‘very high’ risk (Almino Afonso), totalling 3479 inhabitants (0.15% of the analysed population); 74 cities (48%) had a ‘high’ level of risk in the state WSRI, with a total of 373 702 inhabitants (16.75% of the analysed population) in this category; 62 cases (41%) of ‘medium’ risk and 426 401 inhabitants (19% of the analysed population) in this class; 16 cities (10%) were classified as ‘low’ risk of water shortage, totalling 1 442 644 inhabitants (64% of the analysed population). Thus, the category with most cities, and, consequently, the largest portion of the state territory, is the ‘high’ risk. However, this class is not the one with the most urban inhabitants. That goes to the ‘low’ risk category, where the most populated cities of the State are Natal, Mossoró and Parnamirim.

In this context, for the Environmental indicator, problems related to the low outflow and availability of the underground fountainhead (V1), as well as the low availability of superficial water bodies (V2) stand out. These variables also present higher weight in the indicator (1.25). Regarding the Infrastructure indicator, the variable that refers to the time of installation of the urban water distribution network (V7) stands out, with the majority of the cities classified as ‘very high’ risk. Another variable in highlight is the low capacity of the reservoirs located in the cities (V6), with the majority of them registering ‘high’ risk, since storing water in times of scarcity is fundamental for the resistance to the water shortage. In several cities of the State, there is only one reservoir with up to 50 m^3^ of capacity.

Still on this indicator, the type of municipal capture (V5), which has higher weight in the indicator (1.5), registered a large part of the cities with ‘high’ and ‘very high’ risk (30% of the cases), followed by the ‘medium’ risk class in 25% of the cases. Therefore, the risk for this indicator in the State increased, since many cities capture water in large or small local dams in the Potiguar crystalline that have low water availability, and, therefore, high vulnerability in times of prolonged dry weather.

In turn, the State Planning indicator had as a highlight the variables that refer to the absence or precariousness of the municipal risk management (V14) and structural measures for the reduction of the risk of shortage, such as structural construction works or projects (V13). Besides these, for the variable regarding occurrences of water collapse in the city (V11), 45 cases of ‘very high’ risk were found, namely, where occurrences registered in the last period of drought (2012–2017) took place. This variable has higher weight in the indicator, which directed its concerning result, with ‘high’ and ‘very high’ risk, especially for the regions of Saridó (87% of the cases) and Alto Oeste (81% of the cases).

Lastly, there is the Social and Economic indicator, where the variables referring to the income (V16) and education (V17) stood out negatively. In these variables, the majority of the cities of the State were classified as ‘high’ and ‘very high’ risk, revealing the problems that the inhabitants would have to face, resist, and adapt in a situation of water shortage disaster. In this context, Figure 2 shows the configuration of the result of the WSRI in regards to the 19 variables, separately, and Figure 3 shows the general result of the WSRI for the state of RN alongside the results of the indicators.

Figure 3 allows us to identify that the State had the majority of the cities with ‘high’ risk of municipal water shortage risk, with distributed cases, especially in the Seridó and Alto Oeste regions. These two regions were also the ones that most had occurrences of cities in collapse of water supply, demonstrating the higher vulnerability to the water shortage of these regions, highlighting mainly the environmental factor. In this context, the variables related to the climate and availability of superficial water resources are the most determinant for the increase of vulnerability, and, in this way, of the risk of water shortage in these regions. Therefore, after the analysis of the results, it became necessary to propose structural measures for the mitigation of risk of disaster in RN, which complements the analysis of the WSRI in this research.

After the ‘high’ risk class, the ‘medium’ risk stood out in the result of the WSRI of RN. This class can be characterised by the reduction of the vulnerability of the cities through the reduction of the exposition and increase of capacity of coping from the cities, be it through a higher water infrastructure that they have available, low quantity of people, and/or environmental characteristics that allow for a higher safety for water supply. The regions with more occurrences of ‘medium’ risk were the Agreste (region of transition between the coast and semi-arid) and Western regions, where most cities serviced by the water supply networks with capture on the Potiguar basin (and Pernambuco-Paraíba basin) are also cities with relatively low population contingent. In what concerns the Environmental indicator, in the Agreste region, the climate is less dry and there is more superficial water availability than in the West, which also contributed for the result.

## Conclusion

The assessment of the risk of water shortages, its dissemination and the proposal of mitigating measures, which structure this article, are in line with the objectives of DRR advocated both by SEDEC – National Secretariat for Civil Protection and Defense of Brazil, government agency linked to the MDR – Ministry of Regional Development, which has projects financed by the UNDP (United Nations Development Programme), such as the Project for the Improvement of Risk and Disaster Management in Brazil. Projects like this follow the cooperation between SEDEC and UNDP, signed in 2012, which establishes good practice actions that help the country achieve international goals such as the Sustainable Development Goals (SDGs) and the Sendai Framework.

The work presented here is directly related to SDG 6 – Ensuring the availability and sustainable management of water and sanitation for all – as well as SDG 11 – Making cities and human settlements inclusive, safe, resilient and sustainable, mainly on the goal of implementing integrated policies and plans for climate change mitigation and adaptation, disaster resilience in line with the Sendai Framework for DRR 2015–2030.

In the Brazilian context, the research fits into the perspective of the objectives of the National Policy on Water Resources (PNRH, Law number 9.433/1997), specifically when it brings in its Article 2 that ‘the objectives of the National Policy on Water Resources are: I – ensure to current and future generations the necessary availability of water, in quality standards appropriate to the respective uses; II – the rational and integrated use of water resources, including water transport, with a view to sustainable development; III – the prevention and defense against critical hydrological events of natural origin or resulting from the inadequate use of natural resources’.

The results of this research can contribute directly to the management of water resources of the State, diagnosing problems, such as the identification of most vulnerable cities and what indicators must be improved and suggesting measures for the reduction of the vulnerability of the cities, through the improvement of the capacity of coping, susceptibility, and adaptation of the population from the State.

Generally speaking, the result of the WSRI is largely because of the environmental characteristics that increase the exposition to water shortage, as well as the problems found regarding the water infrastructure (especially related to the type of municipal water capture), state planning (especially occurrences of water collapse and the lack of investment in sanitation plans and civil defence), and social and economic indicators (especially problems with education and income of the city population), which affect the vulnerability of the population of these cities that compose the territories of risk of these regions of the State in regards to this type of disaster.

In this context, the methodology of analysis of risk of shortage showed to be pertinent and efficient in the quantification and/or qualification of the risk of this type of disaster in a regional level. The intention of using it in other states of Brazil can be carried out, with the variables possibly adapted to the availability of data in each state.
